# Three Patients with COVID-19 and Pulmonary Tuberculosis, Wuhan, China, January–February 2020

**DOI:** 10.3201/eid2611.201536

**Published:** 2020-11

**Authors:** Zhi Yao, Junbo Chen, Qianli Wang, Weiyong Liu, Qi Zhang, Jing Nan, Hai Huang, Yuying Wu, Lan Li, Lu Liang, Lei You, Yingle Liu, Hongjie Yu

**Affiliations:** Wuhan Pulmonary Hospital, Wuhan, China (Z. Yao, J. Nan, H. Huang, Y. Wu, L. Li);; School of Public Health, Fudan University, Key Laboratory of Public Health Safety, Ministry of Education, Shanghai, China (J. Chen, Q. Wang, L. You, H. Yu);; Tongji Hospital, Tongji Medical College, Huazhong University of Science and Technology, Wuhan (W. Liu);; Wuhan University, State Key Laboratory of Virology, Wuhan (Q. Zhang, Y. Liu);; Sichuan University, Chengdu, China (L. Liang)

**Keywords:** 2019 novel coronavirus disease, coronavirus disease, COVID-19, severe acute respiratory syndrome coronavirus 2, SARS-CoV-2, viruses, respiratory infections, zoonoses, pulmonary tuberculosis, tuberculosis and other mycobacteria, co-infection, Wuhan, China

## Abstract

During January–February 2020, coronavirus disease (COVID-19) and tuberculosis were diagnosed for 3 patients in Wuhan, China. All 3 patients had COVID-19 pneumonia. One severely ill patient died after acute respiratory distress syndrome developed. Clinicians and public health officials should be aware of underlying chronic infections such as tuberculosis in COVID-19 patients.

The leading cause of death from a single infectious agent is tuberculosis (TB) (*1*). Globally, an estimated 1.7 billion persons are infected with *Mycobacterium tuberculosis* (*2*), and a country with one of the highest TB burdens in the world is China (*2*,*3*). Co-infection with severe acute respiratory syndrome coronavirus (*4*,*5*) or Middle East respiratory syndrome coronavirus (*6*) and *M. tuberculosis* has been associated with intensive care unit admission. As severe acute respiratory syndrome coronavirus 2 (SARS-CoV-2) emerges, we report 2 patients with COVID-19 and laboratory-confirmed TB and 1 with COVID-19 and clinically diagnosed TB in China.

Patient 1 was a 50-year-old man who became ill with fever and productive cough on December 25, 2019. Pulmonary TB had been diagnosed for this patient 20 years ago, for which he received anti-TB treatment for 6 months (Table; Appendix). At hospital admission, chest auscultation detected bilateral rhonchi and wet rales. While hospitalized, the patient experienced continuous fever, respiratory distress, and hypoxia. A computed tomography (CT) scan of his chest showed bilateral emphysema, bullous cysts, and right pleural effusion. The pleural effusion contained elevated concentrations of adenosine deaminase (ADA) and lactate dehydrogenase (LDH), and results of a Rivalta test for pleural effusion and *M. tuberculosis* DNA tests were positive. Sputum samples were positive for acid-fast bacilli (AFB) and for *M. tuberculosis* DNA and RNA. On January 28, 2020, a chest CT scan showed progression of bilateral patchy ground-glass opacities (Appendix). The diagnosis was severe pneumonia, laboratory-confirmed active pulmonary TB, anemia, and hypoproteinemia. The patient received anti-TB and corticosteroid treatments and oxygen therapy. The patient’s dyspnea gradually deteriorated; subsequently, acute respiratory distress syndrome developed. On January 29, he died of respiratory and circulatory failure; a throat swab sample taken that day was positive for SARS-CoV-2 RNA.

Patient 2 was a 44-year-old man who became ill with fever, fatigue, headache, and dry cough on January 16. Chest CT scan showed bilateral patchy ground-glass opacities and pleural effusion (Appendix). On February 14, a chest CT scan showed signs of cavitation, which according to the patient’s medical records were new, and a throat swab sample tested positive for SARS-CoV-2 RNA. At admission, the patient had tachycardia. A TB purified protein derivative skin test showed an induration of 7 × 10 mm. Rivalta test was positive for pleural effusion, which contained elevated concentrations of C-reactive protein, LDH, and ADA (35.1 U/L) and was infiltrated with lymphocytes. Sputum and pleural effusion AFB smears were negative. The clinical diagnosis was active pulmonary TB, tuberculous pleuritis, and pleural effusion. While hospitalized, the patient received antiviral drugs and a fixed-dose combination of isoniazid, rifampicin, pyrazinamide and ethambutol. After admission, the patient was found to have type 2 diabetes mellitus, for which acarbose and metformin were prescribed. His signs and symptoms improved after treatment, and he was discharged on March 3 with anti-TB treatment to be continued.

Patient 3 was a 57-year-old man with a 3-year history of diabetes mellitus who on January 16 became ill with cough. In 2001, pulmonary TB had been diagnosed and considered cured. On January 27, 2020**,** according to the patient’s medical records, a chest CT scan showed signs of TB. On February 3, another CT scan showed bilateral patchy ground-glass opacities (Appendix). On February 5, the patient was transferred to Wuhan Pulmonary Hospital, Wuhan, China, where a test for SARS-CoV-2 was positive. At admission, the patient had tachypnea and a peripheral capillary oxygen saturation of 90%. His sputum was positive for *M. tuberculosis* DNA. The diagnosis was severe COVID-19 pneumonia and latent pulmonary TB. While hospitalized, the patient received antibiotics, antiviral drugs, corticosteroids, and oxygen support. On February 7, a chest CT scan showed progression of the ground-glass opacities. Immunoglobulin was administered. Additional CT scans showed gradual improvement, and the patient was discharged on March 2.

All 3 patients with SARS-CoV-2 infection and pulmonary TB had COVID-19 pneumonia; illness was moderate for 1 patient and severe for the other 2. The patient in whom acute respiratory distress syndrome developed died of respiratory and circulatory failure. 

In consideration of the high disease burden of TB and the rapid spread of COVID-19, the potential effects of a possible interaction between the 2 infections requires attention (*7*; P. Glaziou, unpub. data, https://www.medrxiv.org/content/10.1101/2020.04.28.20079582v1). As for general COVID-19 patients, the spectrum of disease for COVID-19 patients with TB can vary from moderate to severe respiratory illness and even death. Underlying conditions including chronic obstructive pulmonary disease, diabetes, hypertension, and malignancy have been associated with more severe outcomes in COVID-19 patients (*8*). However, in our study, the outcome for 1 of the 3 co-infected patients was severe despite his having no other known conditions thought to predispose him to severe COVID-19. Clinicians and public health officials should remain aware of heightened risks caused by chronic infections such as TB in COVID-19 patients.

**Table:** Summary of clinical characteristics and clinical laboratory results for 3 patients with coronavirus disease and tuberculosis, Wuhan, China, January–February 2020*

Characteristics	Patient 1	Patient 2	Patient 3
Age, y	50	44	57
Smoker	+	+	–
Underlying medical conditions†	–	+	+
Signs and symptoms‡			
Fever	+	+	+
Cough	+	+	+
Fatigue	+	+	+
Wheeze	+	+	+
Chills	–	+	–
Weight loss	+	+	+
Night sweats	NA	+	–
Vomiting	–	–	–
Diarrhea	–	–	–
Tachycardia	–	+	–
Tachypnoea	+	–	+
Laboratory findings§			
Leukocyte count, × 10^9^ cells/L (reference range 3.5–9.5 × 10^9^ cells/L)	10.4 (↑)	6.86	8.86
Neutrophil count, ×10^9^ cells/L (reference range 1.8–6.3 × 10^9^ cells/L)	8.74 (↑)	4.35	7.74 (↑)
Lymphocyte count ×10^9^ cells/L (reference range 1.1–3.2 × 10^9^ cells/L)	0.73 (↓)	1.75	0.74 (↓)
T-cell count, cells/μL (reference range 690–2,540 cells/μL)	NA	1,092.92	282.12 (↓)
CD4+ T-cell percentage (reference range 40%–57%)	NA	25.3 (↓)	25.91 (↓)
CD4+ T-cell count, cells/μL (reference range 410–1,590 cells/μL)	NA	415.51	138.91 (↓)
CD8+ T-cell percentage (reference range 8%–37%)	NA	36.67	19.37
CD8+ T-cell count, cells/μL (reference range 190–1,140 cells/μL)	NA	602.14	103.8 (↓)
CD4+ to CD8+ T-cell count ratio (reference range 0.71–2.78)	NA	0.69 (↓)	1.34
Hemoglobin, g/L (reference range 130–175 g/L)	83 (↓)	127 (↓)	134
Platelet count, ×10^9^/L (reference range 125–350 10^9^/L)	430 (↑)	280	218
Activated partial thromboplastin time, s (reference range 27–45 s)	45.5 (↑)	36.4	30.8
Prothrombin time, s (reference range 11–16 s)	18.4 (↑)	13.3	16.29
International normalized ratio (reference range 0.8–1.3)	1.44 (↑)	1.03	1
Fibrinogen, g/dL (reference range 2–4 g/dL)	8.01 (↑)	4.5 (↑)	5.07 (↑)
D–dimer, μg/L (reference range 0–0.5 μg/L)	1.58 (↑)	3.7 (↑)	NA
Alanine aminotransferase, U/L (reference range 9–50 U/L)	9	12	52 (↑)
Aspartate aminotransferase, U/L (reference range 15–40 U/L)	3 (↓)	11 (↓)	45 (↑)
Albumin, g/L (reference range 35–55 g/L)	29.9 (↓)	36.7	35.6
Bilirubin, μmol/L (reference range 0–21 μmol/L)	2.56	5	4.15
Creatinine, μmol/L (reference range 44–115 μmol/L)	38 (↓)	NA	64
Lactate dehydrogenase, U/L (reference range 106–245 U/L)	170	NA	367 (↑)
Creatine kinase, U/L (reference range 24.0–194.0 U/L)	31	24.7	45.1
Creatine kinase isoenzyme, U/L (reference range 0–24 U/L)	9	12.6	22.6
Bicarbonate, mmol/L (reference range 22–27 mmol/L)	21.4	29.9 (↑)	29.8 (↑)
C-reactive protein, mg/L (reference range 0.0–5.0 mg/L)	293.8 (↑)	3.99	44.4 (↑)
Procalcitonin, ng/mL (reference range 0.00–0.25 ng/mL)	0.14	0.04	0.04
Erythrocyte sedimentation rate, mm/h; (reference range 0–15 mm/h)	123 (↑)	81 (↑)	53 (↑)
Chest CT findings			
Ground-glass opacities	+	+	+
Pleural effusion	+	+	–
Treatment			
Antibiotics	+	+	+
Anti-TB therapy	+	+	–
Lopinavir/ritonavir	–	+	+
Umifenovir hydrochloride	–	+	–
Interferon–α	–	+	+
Corticosteroid	+	–	+
Immunoglobulin	–	–	+
Oxygen support	+	–	+
Clinical course			
Duration of hospitalization, d	22	26	27
Time from illness onset to discharge or death, d	35	47	46
Clinical severity	Severe	Moderate	Severe
Outcome	Died	Survived	Survived

*All patients were male. CT, computed tomography; NA, not available; TB, tuberculosis; ↑, values higher than reference range; ↓, values lower than reference range.; +, positive; –, negative. †Patients 2 and 3 had type 2 diabetes mellitus. ‡At admission to the original hospital. §Test results after transfer to the Wuhan Pulmonary Hospital.

Appendix Supplemental methods and results for report of 3 patients with pulmonary tuberculosis and COVID-19, Wuhan, China, January–February 2020.
