# Supplementary material for: Three Patients with COVID-19 and Pulmonary Tuberculosis, Wuhan, China, January–February 2020
Source: Emerg Infect Dis. 2020 Nov;26(11):2754–7. doi: 10.3201/eid2611.201536 (PMC7588523; doi:10.3201/eid2611.201536)
Supplement: Appendix — Supplemental methods and results for report of 3 patients with pulmonary tuberculosis and COVID-19, Wuhan, China, January–February 2020. [file 20-1536-Techapp-s1.pdf]

Article DOI: https://doi.org/10.3201/eid2611.201536

Three Patients with COVID-19 and Pulmonary Tuberculosis, Wuhan, China, January–February 2020

Appendix

Methods

Study Design and Participants

In early 2000, China launched its hospital-based management model for TB, mandating tuberculosis-specific hospitals to diagnose and treat TB patients (*1*). In the region of Wuhan, the Wuhan Pulmonary Hospital (also known as Wuhan Tuberculosis Control Institute) and the Wuhan Jinyintan Hospital are the two designated hospitals for the treatment of TB patients. Due to the outbreak of COVID-19, Wuhan Jinyintan Hospital was converted to provide surge medical care of COVID-19 on January 3, 2020. Since then, the Wuhan Pulmonary Hospital is the only hospital providing medical care for TB patients.

Wuhan Pulmonary Hospital was one of the first COVID-19 designated hospitals. For suspected COVID-19 patients presenting to outpatient departments, or admitted to Wuhan Pulmonary Hospital, respiratory specimens were collected and tested for SARS-CoV-2. Laboratory-confirmed COVID-19 patients who had clinically diagnosed or laboratory-confirmed TB were admitted to Wuhan Pulmonary Hospital, and patients initially hospitalized in other hospitals in Wuhan were transferred. Between January 3 and March 3, 2020, all COVID-19 cases who had clinically diagnosed or laboratory-confirmed TB and were hospitalized in Wuhan Pulmonary Hospital were enrolled in this study.

Diagnoses of Pulmonary tuberculosis and COVID-19

The diagnosis of pulmonary tuberculosis (PTB) was made based on clinical presentation, chest radiography, and laboratory investigation, according to the diagnostic guidelines for PTB issued by the National Health Commission (NHC) of China (*2*). An adult was clinically diagnosed with PTB if the following clinical criteria were met:

i) typical radiographic findings indicating a high clinical probability of PTB;

ii) suspicious symptoms (cough for >2 weeks, with or without night sweats, fatigue, weight loss, chest pain, dyspnea) or signs (tubular breath sound or wet rales on chest auscultation) of PTB, or tuberculosis purified protein derivative (PPD) skin test positive.

Patients with typical radiographic findings indicating PTB and laboratory detected *Mtb* in their specimens (culture or nucleic acid amplification testing) were classified as laboratory-confirmed PTB cases. Suspected and laboratory-confirmed COVID-19 cases were defined according to the guidelines for COVID-19 issued by China NHC (*3*).

Procedure and Data Collection

Demographic, clinical, laboratory and radiological data were reviewed from medical records. Data available included detailed information on comorbidities, clinical presentation, complications, and treatments for both COVID-19 and TB. The date of illness onset was defined as the day when COVID-19 symptoms were first noticed and was interpreted as “day 0”.

Ethics

The study protocol was reviewed and approved by the ethics committee of Wuhan Pulmonary Hospital. Written informed consent was obtained from each patient.

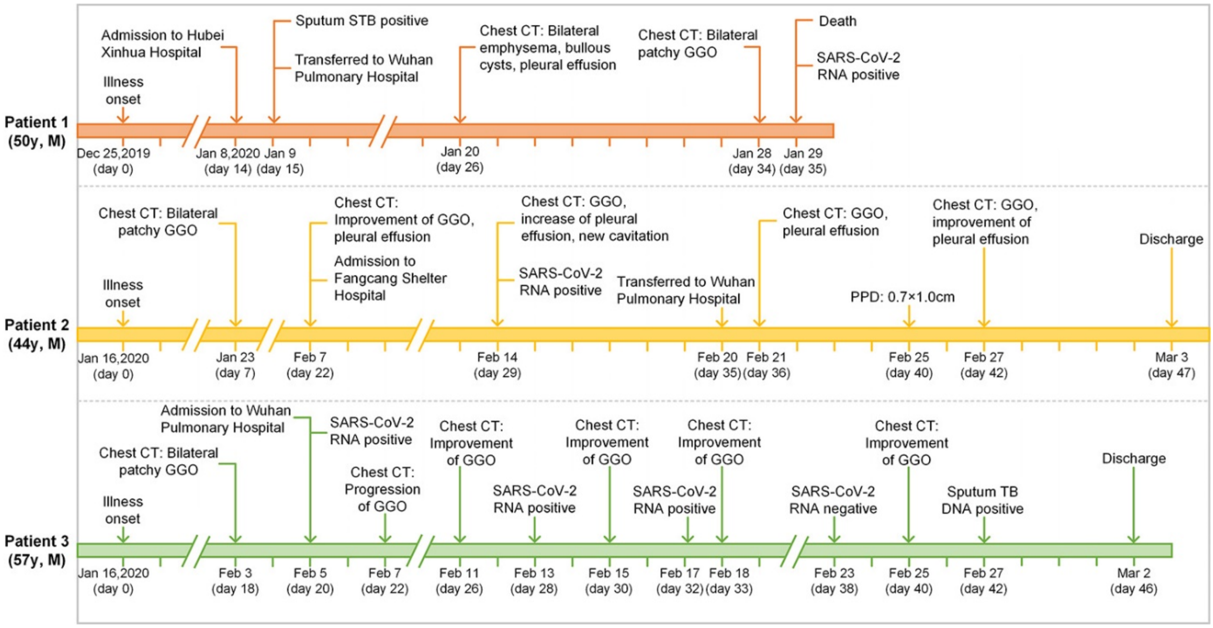

Appendix Figure 1. The clinical course of three patients with SARS-CoV-2 and TB coinfections.

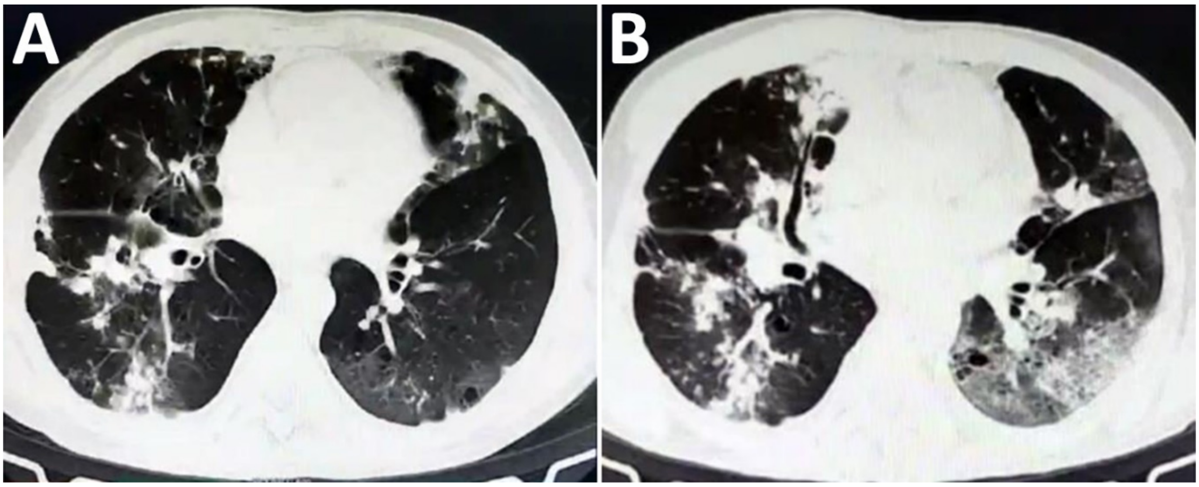

Appendix Figure 2. Chest CT scan images of patient 1. Representative chest CT scan images on January 20 and January 28, both were taken during the patient’s hospitalization in Wuhan Pulmonary Hospital.

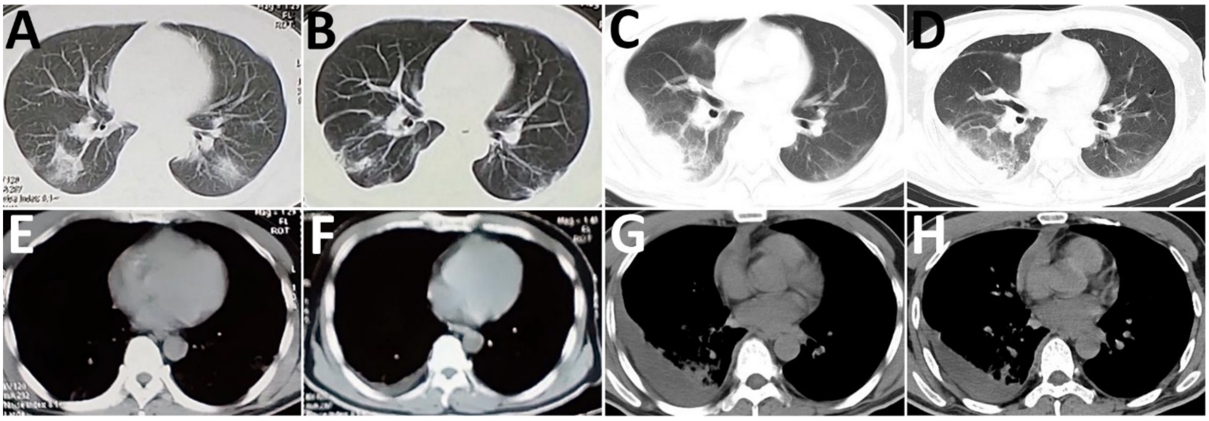

Appendix Figure 3. Chest CT scans of patient 2. Representative chest CT scan images showing bilateral patchy ground-glass opacities (GGO) in the lung on January 23, and gradual improvement of GGO on February 7, 21, and 27 (upper panels). Representative chest CT scans showing pleural effusion in the lung on February 7, increased pleural effusion on February 21, and improvement on February 27 (lower panels). CT scans were performed in Huangpi District People's Hospital on January 23, in Wuhan Jinyintan Hospital on February 7, and in Wuhan Pulmonary Hospital on February 21 and 27.

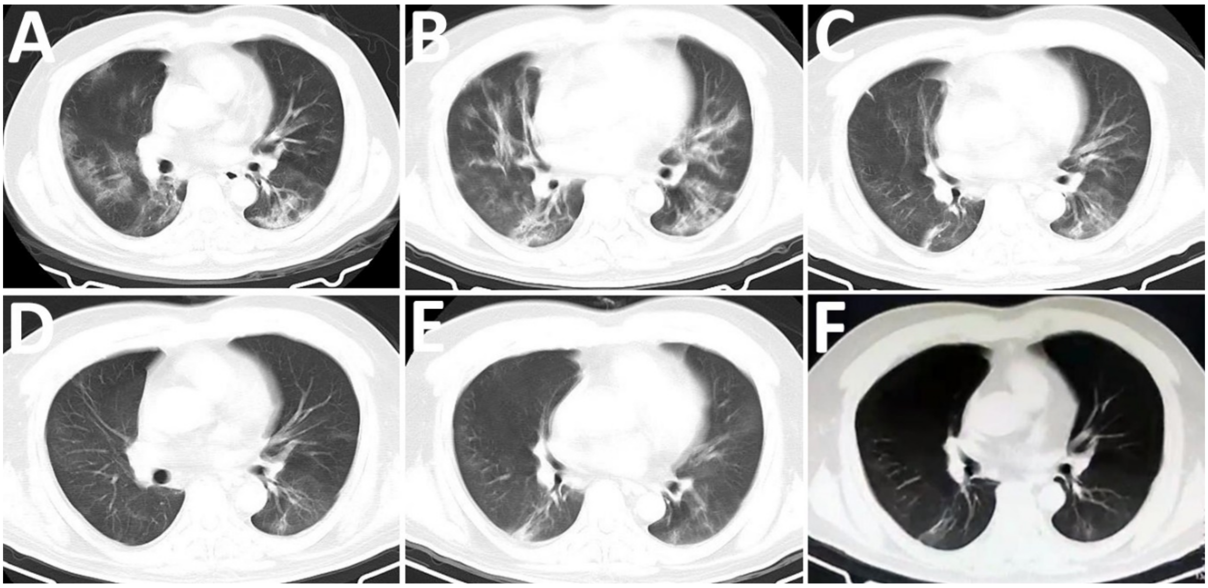

Appendix Figure 4. Chest CT scans of patient 3. Representative chest CT scans showing bilateral patchy ground-glass opacities (GGO) in the lung on February 3, a progression of GGO on February 7 and gradual improvement of GGO on February 11, 15, 18, and 25. All CT scans were performed in Wuhan Pulmonary Hospital.

References

1. Pang Y, Du J, Qin ZZ, Greenwald Z, Liu Y, Mi F, et al. An overview on tuberculosis-specific hospitals in China in 2009: results of a national survey. Eur Respir J. 2016;47:1584–7. PubMed https://doi.org/10.1183/13993003.01854-2015

2. National Health Commission. Diagnosis for pulmonary tuberculosis WS 288-2017 (in Chinese) [cited 2020 Apr 6]. http://www.nhc.gov.cn/ewebeditor/uploadfile/2017/11/20171128164254246.pdf

3. National Health Commission. Diagnosis and treatment protocol for novel coronavirus pneumonia (trial version 7) [cited 2020 Apr 6]. https://www.who.int/docs/default-source/wpro---documents/countries/china/covid-19-briefing-nhc/1-clinical-protocols-for-the-diagnosis-and-treatment-of-covid-19-v7.pdf?sfvrsn=c6cbfba4_2
